# Supplementary material for: Warburg-Cinotti disease variant p.Tyr740Cys enhances catalytic activity of DDR2 kinase
Source: PLoS One. 2025 Nov 19;20(11):e0336895. doi: 10.1371/journal.pone.0336895 (PMC12629418; doi:10.1371/journal.pone.0336895)
Supplement: S1 Table — Mutagenic bases are shown in bold. Flag-tag insertion sequences are shown in bold and underlined. (DOCX) [file pone.0336895.s001.docx]

**S1 Table. Primer List.** Mutagenic bases are shown in bold. Flag-tag insertion sequences are shown in bold and underlined.

| **Primer Name** | **Forward primer (5’-3’)** | **Application** |
| --- | --- | --- |
|  | **Reverse primer (5’-3’)** |  |
| DDR2Y740C | CTGTACAGTGGTGACT**G**TTACCGGATCCAGG | QuickChange Mutagenesis |
|  | CCTGGATCCGGTAA**C**AGTCACCACTGTACAG |  |
| DDR2L610P | GGCTGTGAAAATGC**C**CCGAGCAGATGCC |  |
|  | GGCATCTGCTCGG**G**GCATTTTCACAGCC |  |
| Flag-GS | **GATGACGATAAGAGTGGTTCTGGATCAGGTTCG**CCAGCTATATGCCGCTATCCTC |  |
|  | **GAACCACTCTTATCGTCATCGTCCTTATAGTC**ATTAACCTGAGCTTTTGCAGAACTCA |  |
| pOPIN-DDR2 | AAGTTCTGTTTCAGGGCCCGGTGCCCCACTATGCAGAG | In-Fusion cloning |
