# Supplementary material for: Warburg-Cinotti disease variant p.Tyr740Cys enhances catalytic activity of DDR2 kinase
Source: PLoS One. 2025 Nov 19;20(11):e0336895. doi: 10.1371/journal.pone.0336895 (PMC12629418; doi:10.1371/journal.pone.0336895)
Supplement: S2 Table — GST, glutathione S-transferase; SUMO, small ubiquitin-like modifier; MBP, maltose-binding protein. (DOCX) [file pone.0336895.s002.docx]

**S2 Table. List of pOPIN constructs and their protein constructs names.** GST, glutathione S-transferase; SUMO, small ubiquitin-like modifier; MBP, maltose-binding protein.

| **DNA construct name** | **Tag** | **Protein construct name** |
| --- | --- | --- |
| pOPINF-DDR2-WT |  | DDR2-K-WT |
| pOPINF-DDR2-Y740C | His_6_-tag | DDR2-K-Y740C |
| pOPINF-DDR2-L610P |  | DDR2-K-L610P |
| pOPINJ-DDR2-L610P | His_6_-GST-tag | His-GST-DDR2-K-L610P |
| pOPINS3C-DDR2-WT | His_6_-SUMO-tag | His-SUMO-DDR2-K-WT |
| pOPINS3C-DDR2-L610P |  | His-SUMO-DDR2-K-L610P |
| pOPINM-DDR2-WT | His_6_-MBP-tag | His-MBP-DDR2-K-WT |
| pOPINM-DDR2-L610P |  | His-MBP-DDR2-K-L610P |
