# Supplementary material for: Warburg-Cinotti disease variant p.Tyr740Cys enhances catalytic activity of DDR2 kinase
Source: PLoS One. 2025 Nov 19;20(11):e0336895. doi: 10.1371/journal.pone.0336895 (PMC12629418; doi:10.1371/journal.pone.0336895)
Supplement: S1 Fig — Clustal Omega alignment of partial sequences of the DDR1 (amino acids 536–913) and DDR2 (amino acids 496–855) cytosolic regions. The JM4 regions are coloured in orange, the αC helices are indicated in teal and the A-loops in green. The catalytically important Lys on the β3 strand (L655 in DDR1, L608 in DDR2) and Glu in the αC helix (E572 in DDR1, E625 in DDR2) are indicated in red, and the A-loop middle Tyr (Y796 in DDR1, Y740 in DDR2) are indicated in yellow. The DDR2 residues mutated in Warburg-Cinotti disease (L610 and Y740) are shown by yellow letters highlighted in black. Sequence numbers are shown to the right. ‘*’ (asterisk), fully conserved residue; ‘:’ (colon), conserved substitutions; ‘.’ (period), less well conserved substitution. (DOCX) [file pone.0336895.s003.docx]

**S1 Fig. Sequence alignments of the JM4 region and TKD of DDR1 and DDR2.**

Clustal Omega alignment of partial sequences of the DDR1 (amino acids 536 to 913) and DDR2 (amino acids 496 to 855) cytosolic regions. The JM4 regions are coloured in orange, the αC helices are indicated in teal and the A-loops in green. The catalytically important Lys on the β3 strand (L655 in DDR1, L608 in DDR2) and Glu in the αC helix (E572 in DDR1, E625 in DDR2) are indicated in red, and the A-loop middle Tyr (Y796 in DDR1, Y740 in DDR2) are indicated in yellow. The DDR2 residues mutated in Warburg-Cinotti disease (L610 and Y740) are shown by yellow letters highlighted in black. Sequence numbers are shown to the right. ‘*’ (asterisk), fully conserved residue; ‘:’ (colon), conserved substitutions; ‘.’ (period), less well conserved substitution.
