# Supplementary material for: Warburg-Cinotti disease variant p.Tyr740Cys enhances catalytic activity of DDR2 kinase
Source: PLoS One. 2025 Nov 19;20(11):e0336895. doi: 10.1371/journal.pone.0336895 (PMC12629418; doi:10.1371/journal.pone.0336895)
Supplement: S2 Fig — A. DDR1 Leu657 (in blue) (equivalent to Leu610 in DDR2) forms a hydrophobic contact (~3.6 Å) with JM4 (in orange) residue Val580 (in yellow). Selected side chains are presented in atomic detail. B. Close-up view of the JM4 hairpin region (residue 577–587). The colour scheme is the same as in A. Selected side chains are presented in atomic detail. (DOCX) [file pone.0336895.s004.docx]

**S2 Fig. DDR1-Leu657 (equivalent to Leu610 in DDR2) forms a contact with the JM4 hairpin residue.**

**A.** DDR1 Leu657 (in blue) (equivalent to Leu610 in DDR2) forms a hydrophobic contact (~3.6 Å) with JM4 (in orange) residue Val580 (in yellow). Selected side chains are presented in atomic detail**.**

**B.** Close-up view of the JM4 hairpin region (residue 577 to 587). The colour scheme is the same as in **A**. Selected side chains are presented in atomic detail**.**
