# Supplementary material for: Warburg-Cinotti disease variant p.Tyr740Cys enhances catalytic activity of DDR2 kinase
Source: PLoS One. 2025 Nov 19;20(11):e0336895. doi: 10.1371/journal.pone.0336895 (PMC12629418; doi:10.1371/journal.pone.0336895)
Supplement: S3 Fig — A. Full-length DDR2-WT, DDR2-Y740C, DDR2-L610P were transiently expressed in HEK293 cells. The cells were lysed two days after transfection. The lysates were treated with EndoH (H) or left untreated (-), followed by boiling in sample buffer and analysis by Western Blotting with anti-DDR2 antibody. The positions of the EndoH sensitive, immature biosynthetic precursor forms are indicated by an orange arrow. The positions of the Endo H resistant mature glycoforms are indicated in blue. The positions of molecular weight markers (in kDa) are shown on the left. B. Expression constructs encoding Flag-DDR2-WT, Flag-DDR2-Y740C or Flag-DDR2-L610P were transfected into Cos-7 cells. Cells were subsequently stained with anti-Flag antibody, followed by AlexaFluor 488 anti-mouse IgG1 secondary antibody. Scale bar = 50 μm. Widefield images were acquired using an Olympus BX51 microscope with Simple-PCI acquisition software. C. Expression constructs encoding Flag-DDR2-WT, Flag-DDR2-Y740C or Flag-DDR2-L610P were singly transfected into HEK293 cells. Cells were subsequently stained with anti-Flag antibody, followed by FITC-labelled anti-mouse-Fc antibody and analysed by flow cytometry. The filled grey histograms represent secondary antibody only staining, while the open histograms represent anti-Flag and secondary antibody staining. D. Quantification of mean fluorescence intensity from the flow cytometry experiments. (DOCX) [file pone.0336895.s005.docx]

**S3 Fig. Cell surface expression of Warburg-Cinotti mutants.**

**A**. Full-length DDR2-WT, DDR2-Y740C, DDR2-L610P were transiently expressed in HEK293 cells. Cells were lysed two days after transfection and lysates were treated with EndoH (H) or left untreated (-), followed by boiling in sample buffer and analysis by Western blotting with anti-DDR2 antibody. The positions of the EndoH sensitive, immature biosynthetic precursor forms are indicated by an orange arrow. The positions of the Endo H resistant mature glycoforms are indicated in blue. The positions of molecular weight markers (in kDa) are shown on the left.

**B**. Expression constructs encoding Flag-DDR2-WT, Flag-DDR2-Y740C or Flag-DDR2-L610P were transfected into Cos-7 cells. Cells were subsequently stained with anti-Flag antibody, followed by AlexaFluor 488 anti-mouse IgG1 secondary antibody. Scale bar = 50 μm. Widefield images were acquired using an Olympus BX51 microscope with Simple-PCI acquisition software.

**C**. Expression constructs encoding Flag-DDR2-WT, Flag-DDR2-Y740C or Flag-DDR2-L610P were singly transfected into HEK293 cells. Cells were subsequently stained with anti-Flag antibody, followed by FITC-labelled anti-mouse-Fc antibody and analysed by flow cytometry. The filled grey histograms represent secondary antibody only staining, while the open histograms represent anti-Flag and secondary antibody staining.

**D.** Quantification of mean fluorescence intensity from the flow cytometry experiments.
