# Supplementary material for: Warburg-Cinotti disease variant p.Tyr740Cys enhances catalytic activity of DDR2 kinase
Source: PLoS One. 2025 Nov 19;20(11):e0336895. doi: 10.1371/journal.pone.0336895 (PMC12629418; doi:10.1371/journal.pone.0336895)
Supplement: S4 Fig — Constructs encoding His-tagged DDR2-K-WT, SUMO-tagged DDR2-K-WT, or SUMO-tagged DDR2-K-L610P were transfected into HEK293 cells. The His-tagged proteins were then isolated from the cell lysates with cobalt-based immobilized metal affinity Dynabeads. The purified protein/bead mixtures were then stimulated with 1 mM ATP in kinase buffer I for 30 min at 20°C. Samples were boiled in sample buffer and analysed by SDS-PAGE and Western blotting with the JM4-specific and A-loop-specific anti-pY antibodies, as indicated. Total DDR2 levels were detected using an anti-DDR2 antibody. The positions of molecular weight markers (in kDa) are shown on the left. CL, cell lysate; HP, affinity isolated His-tagged protein. (DOCX) [file pone.0336895.s006.docx]

**S4 Fig. *In vitro* kinase activity of soluble recombinant DDR2 kinase constructs.**

Constructs encoding His-tagged DDR2-K-WT, SUMO-tagged DDR2-K-WT, or SUMO-tagged DDR2-K-L610P were transfected into HEK293 cells. The His-tagged proteins were then isolated from the cell lysates with cobalt-based immobilized metal affinity Dynabeads. The purified protein/bead mixtures were then stimulated with 1 mM ATP in kinase buffer I for 30 min at 20°C. Samples were boiled in sample buffer and analysed by SDS-PAGE and Western blotting with the JM4-specific and A-loop-specific anti-pY antibodies, as indicated. Total DDR2 levels were detected using an anti-DDR2 antibody. The positions of molecular weight markers (in kDa) are shown on the left. CL, cell lysate; HP, affinity isolated His-tagged protein.
