# Supplementary material for: Warburg-Cinotti disease variant p.Tyr740Cys enhances catalytic activity of DDR2 kinase
Source: PLoS One. 2025 Nov 19;20(11):e0336895. doi: 10.1371/journal.pone.0336895 (PMC12629418; doi:10.1371/journal.pone.0336895)
Supplement: S1 File — Uncropped images of all blots shown, lanes labelled and annotated as in the respective figures. (PDF) [file pone.0336895.s007.pdf]

Figure 2

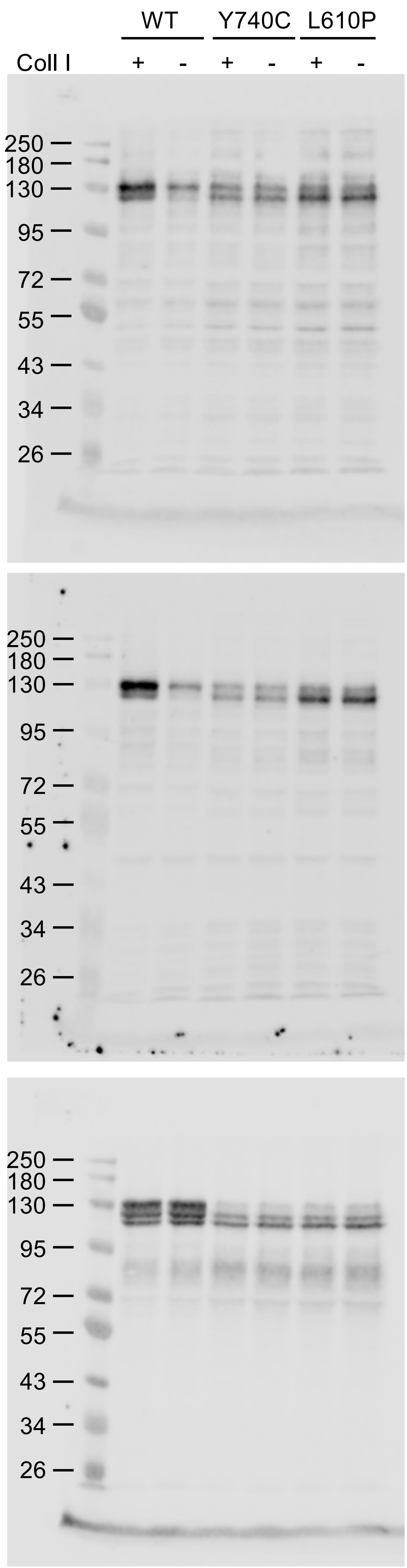

Figure 2A, Panel 1

anti-pY (4G10)

Figure 2A, Panel 2

anti-pY JM4 #1

Figure 2A, Panel 3

anti-DDR2

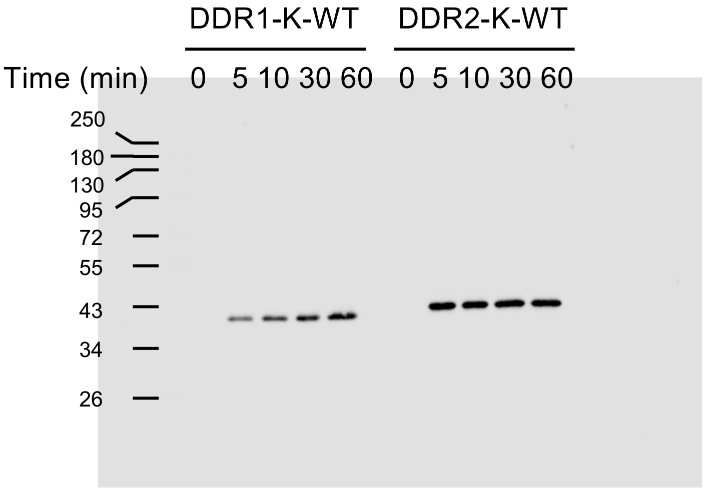

Figure 3B
Panel 1
anti-pY JM4 #1

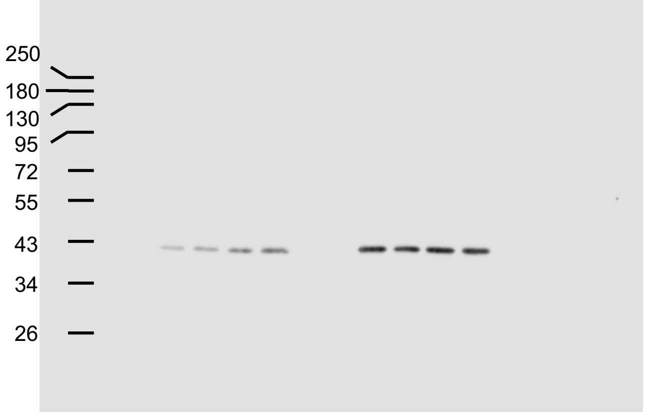

Figure 3B
Panel 2
anti-pY JM4 #2

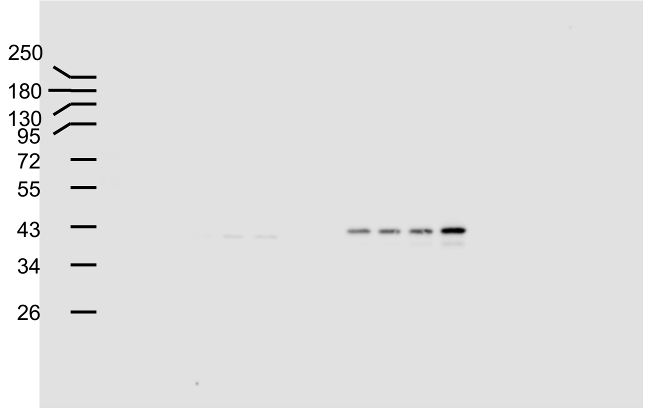

Figure 3B
Panel 3
anti-pY A-loop

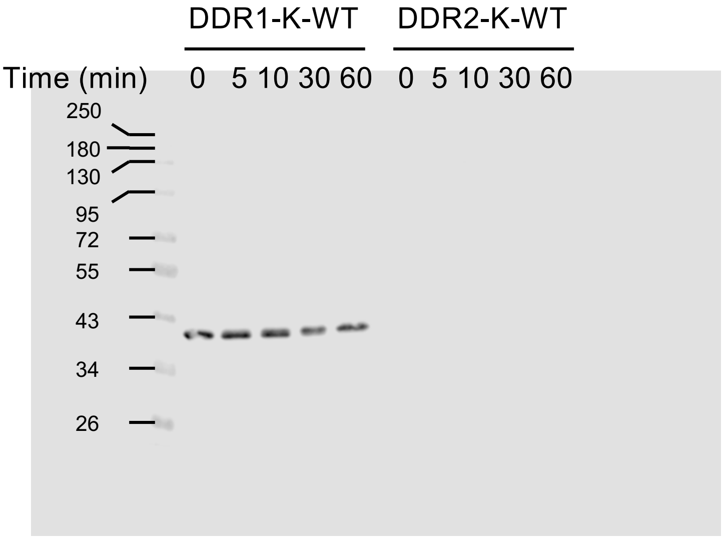

Figure 3B
Panel 4
anti-DDR1

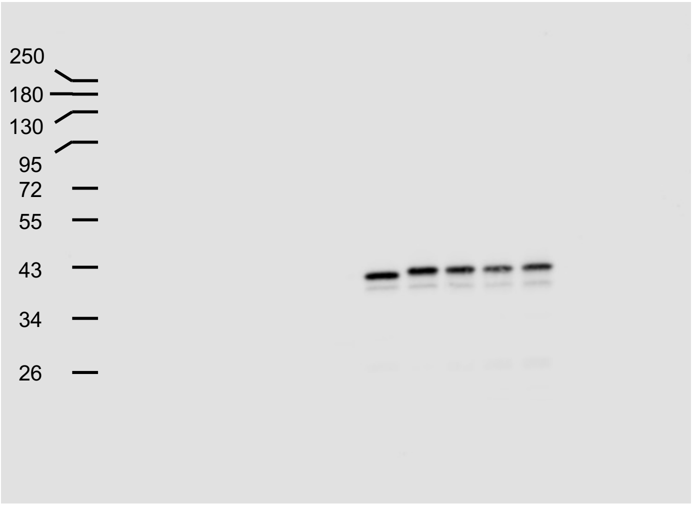

Figure 3B
Panel 5
anti-DDR2

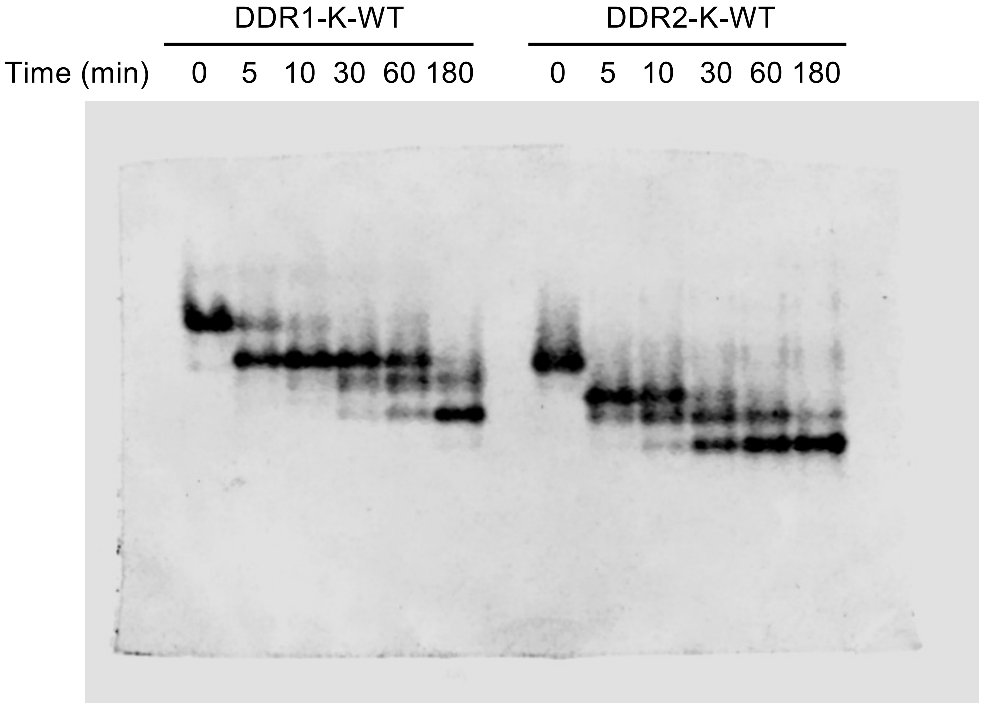

Figure 4
Panel 1
Coomassie stain

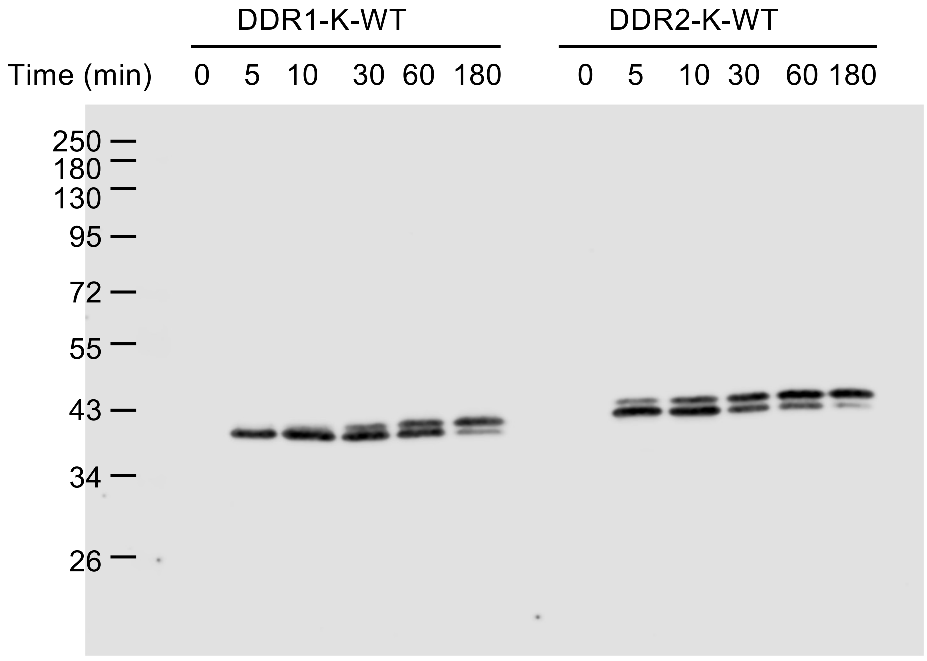

Figure 4
Panel 2
anti-pY JM4 #1

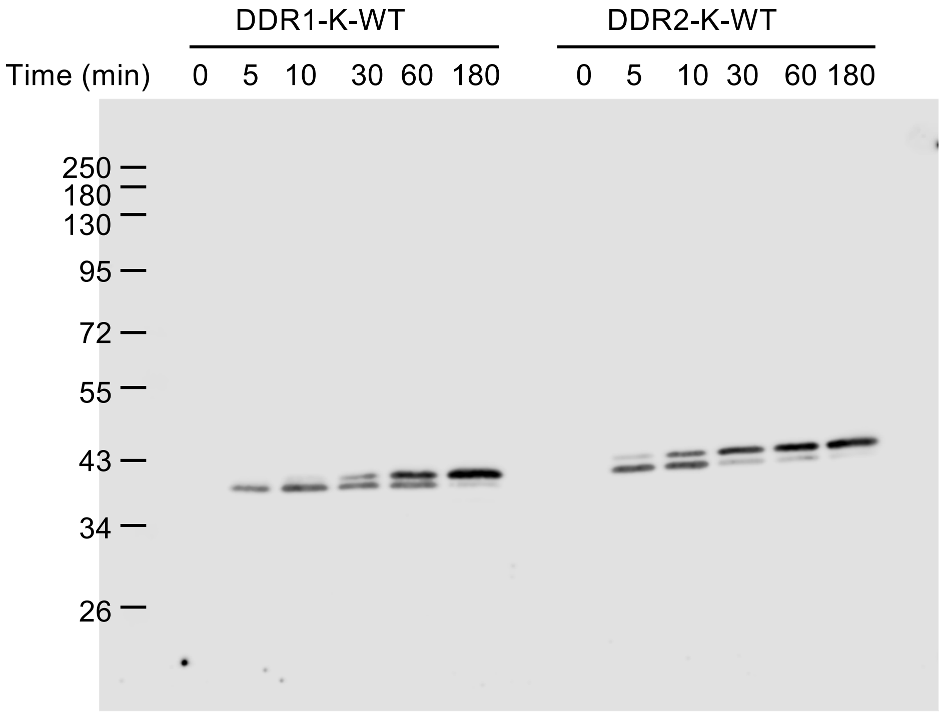

Figure 4
Panel 3
anti-pY JM4 #2

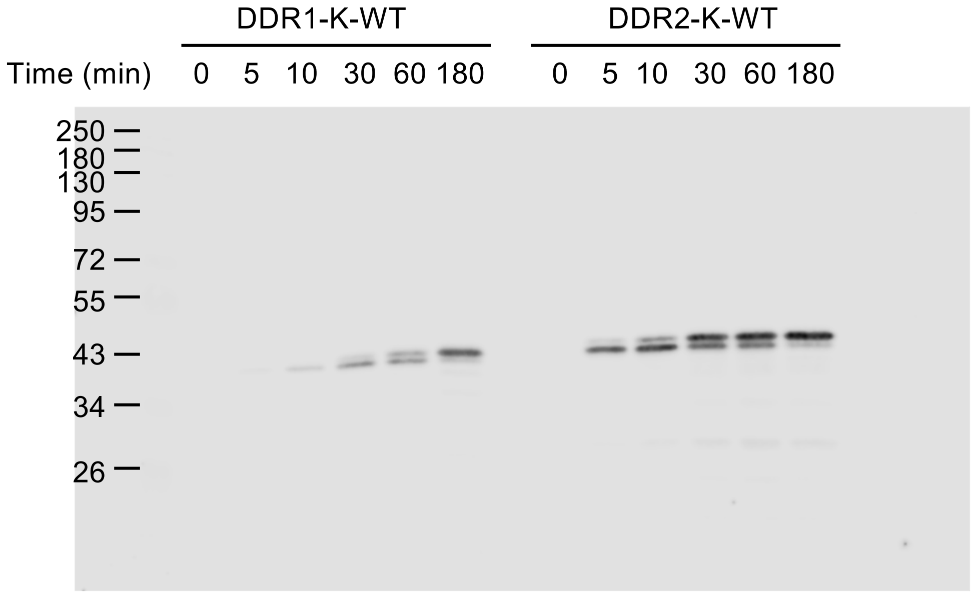

Figure 4
Panel 4
anti-pY A-loop

Figure 4

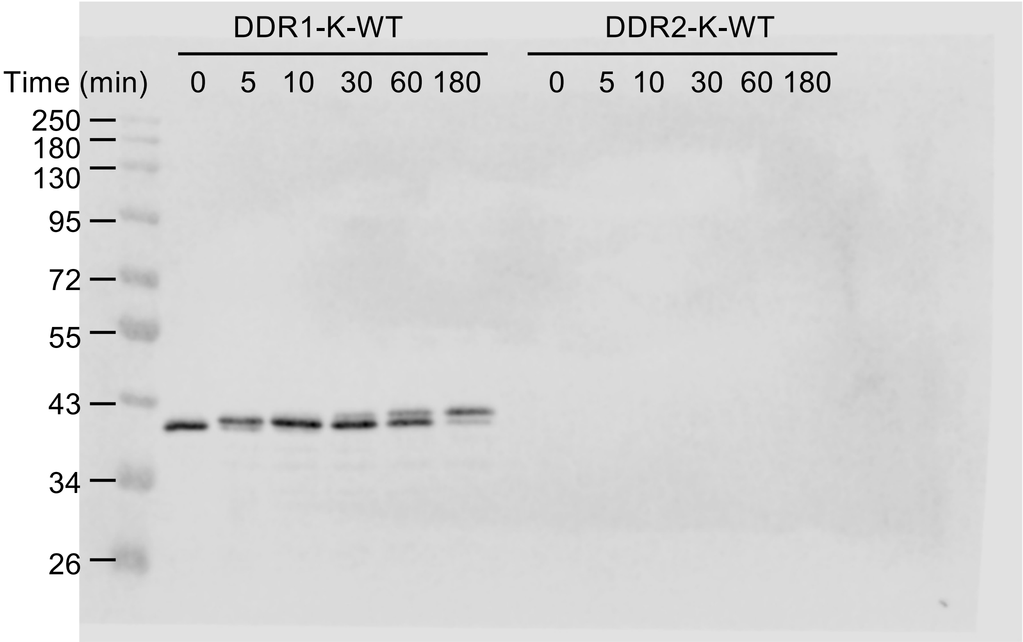

Figure 4
Panel 5
anti-DDR1

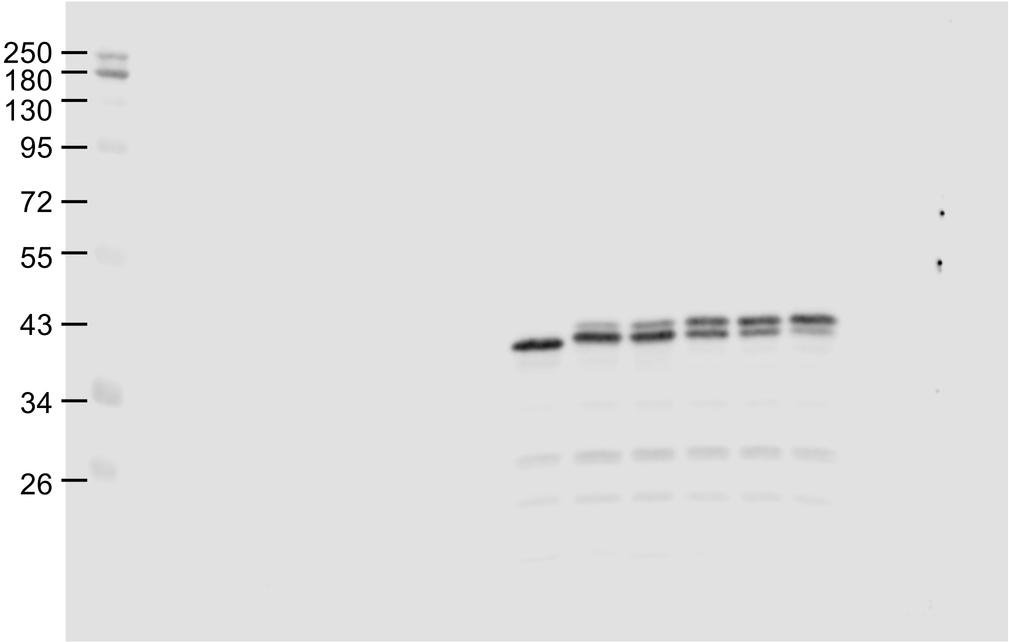

Figure 4
Panel 6
anti-DDR2

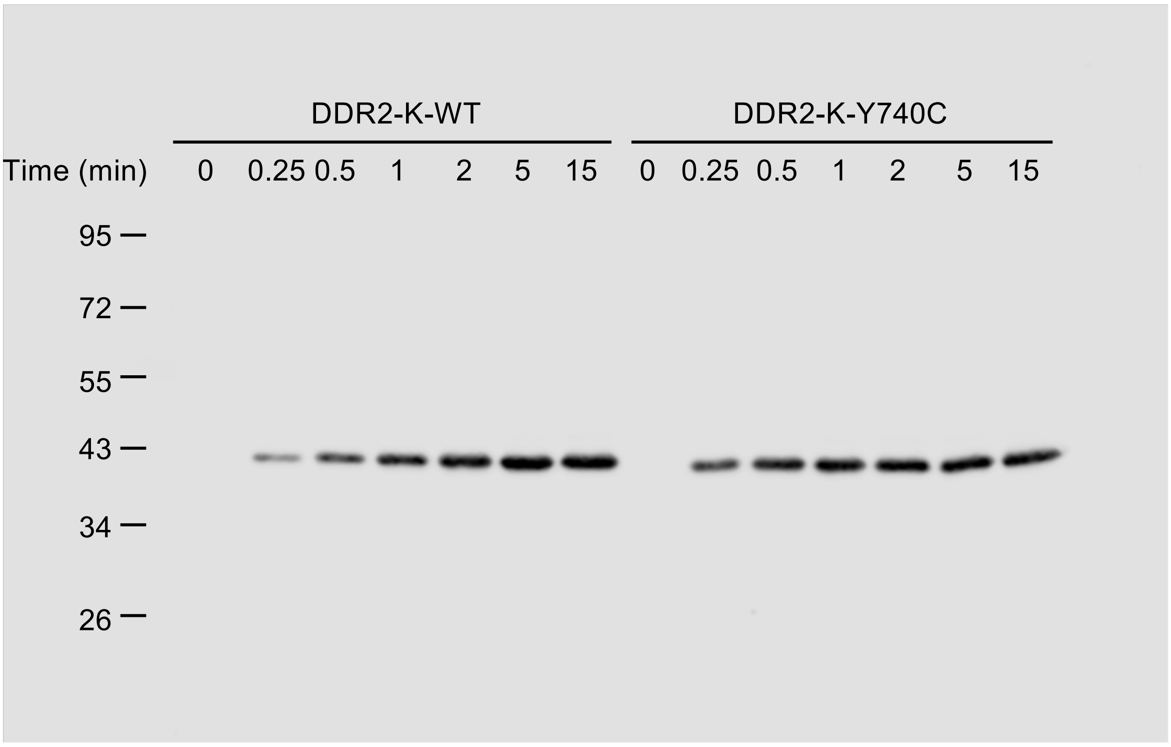

Figure 5
Panel 1
anti-pY JM4 #1

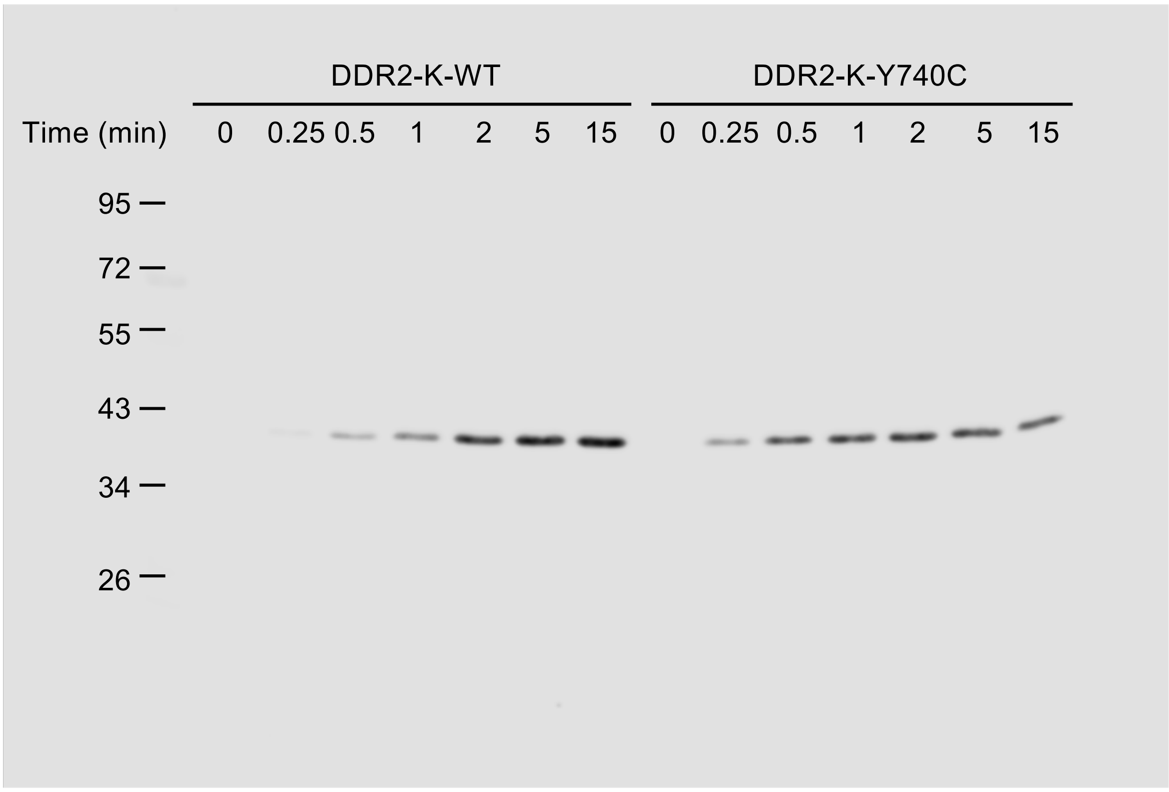

Figure 5
Panel 2
anti-pY JM4 #2

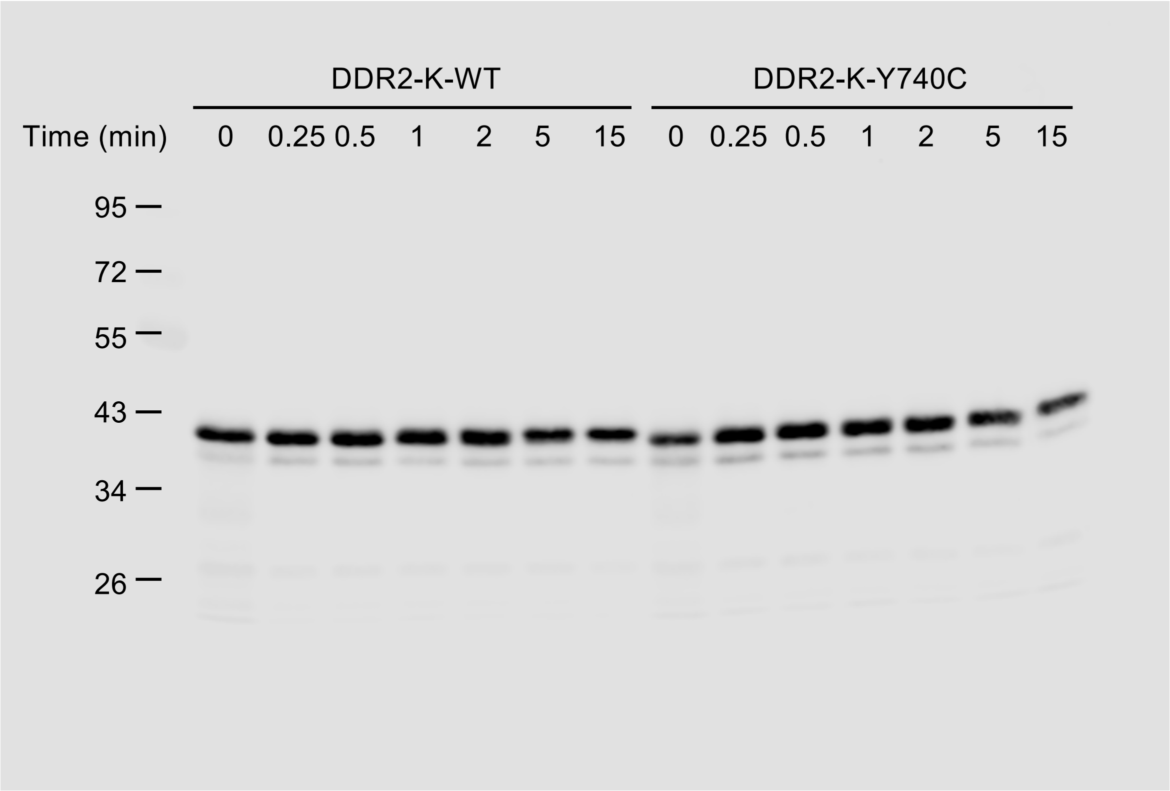

Figure 5
Panel 3
anti-DDR2

Figure 6

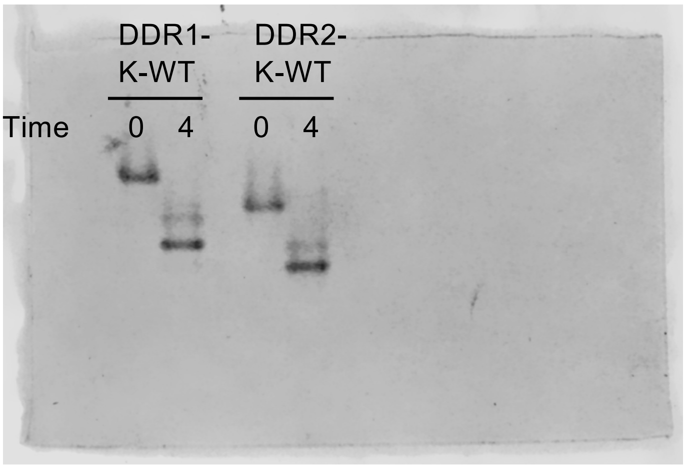

Figure 6
Panel 1

Coomassie stain

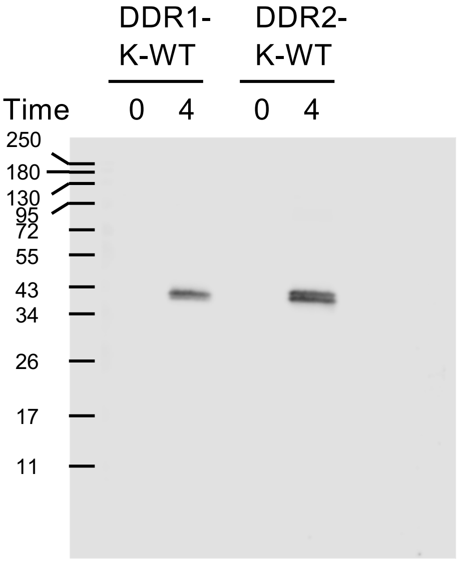

Figure 6
Panel 2

anti-pY JM4 #1

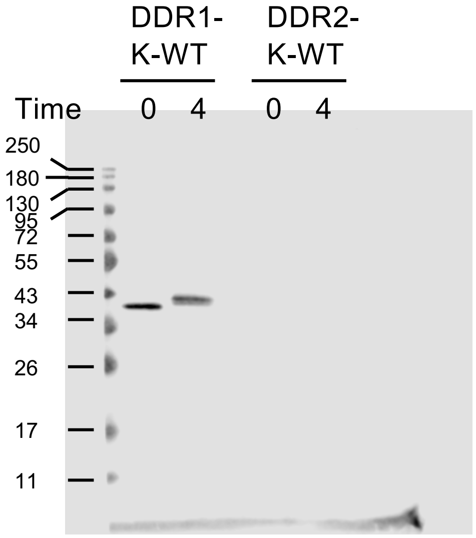

Figure 6
Panel 4
anti-DDR1

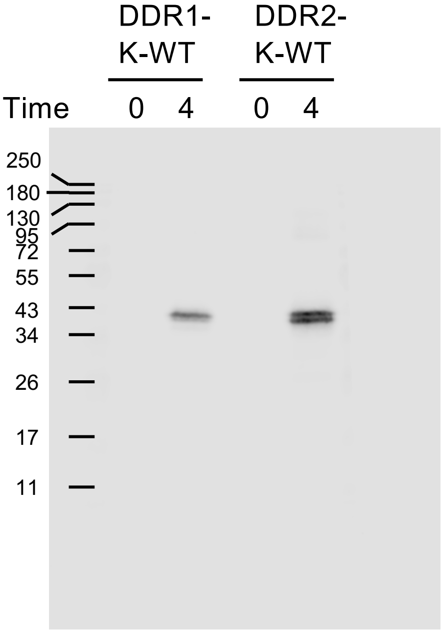

Figure 6
Panel 3

anti-pY A-loop

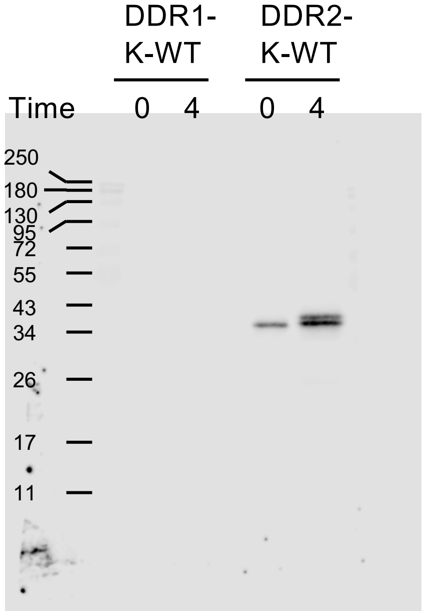

Figure 6
Panel 5
anti-DDR2

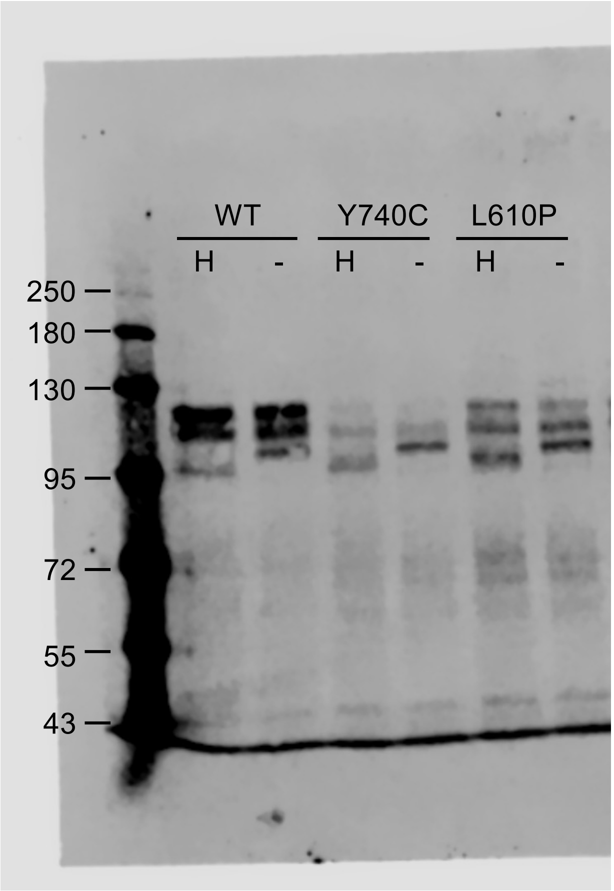

Supporting Figure 3

anti-DDR2

Supporting Figure 4

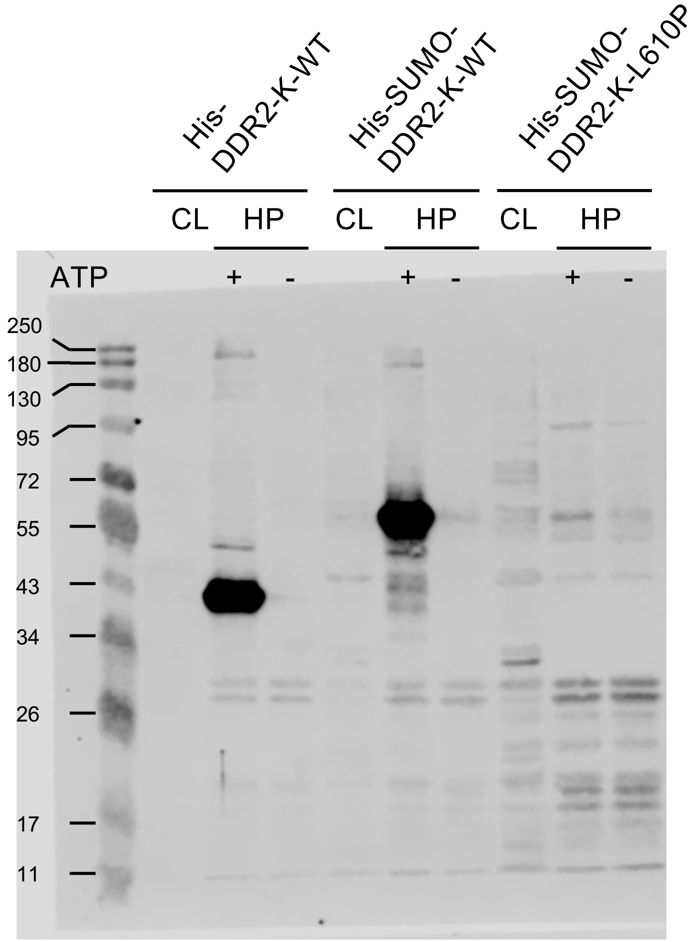

Supporting Figure 4
Panel 1

anti-pY JM4 #1

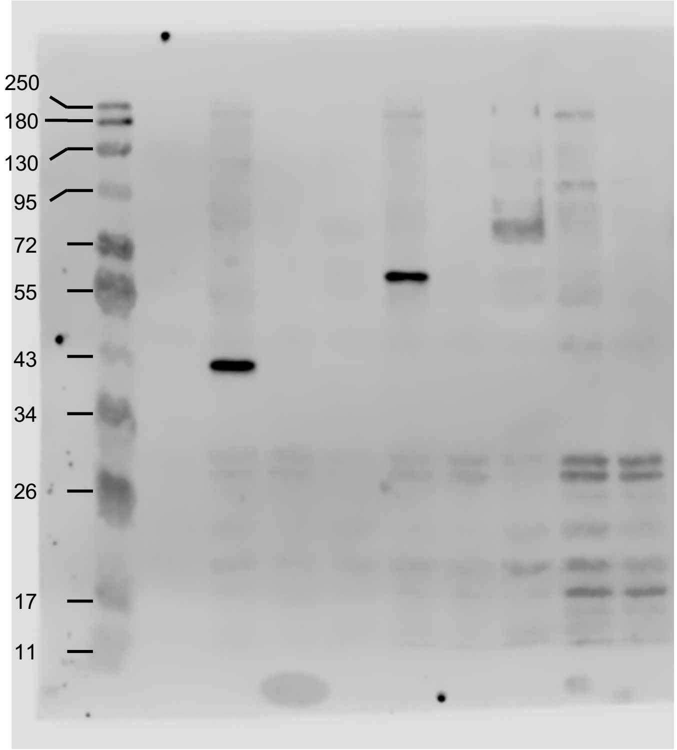

Supporting Figure 4
Panel 2

anti-pY A-loop

Supporting Figure 4

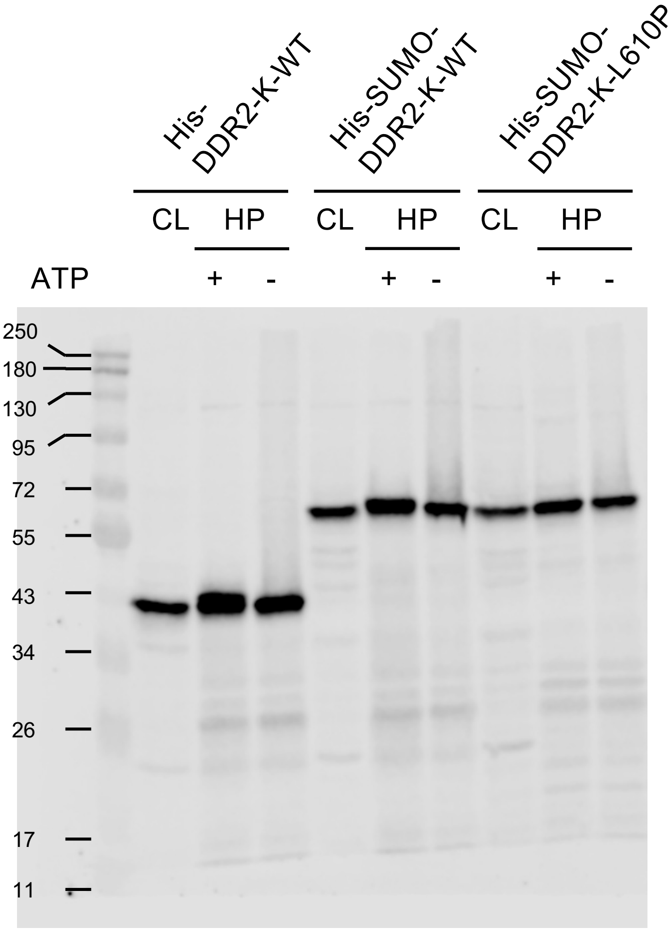

Supporting Figure 4
Panel 3

anti-DDR2
