# Supplementary material for: Warburg-Cinotti disease variant p.Tyr740Cys enhances catalytic activity of DDR2 kinase
Source: PLoS One. 2025 Nov 19;20(11):e0336895. doi: 10.1371/journal.pone.0336895 (PMC12629418; doi:10.1371/journal.pone.0336895)
Supplement: Attachment — Submitted filename: Comments PLos.docx [file pone.0336895.s008.docx]

The manuscript addresses an important topic and presents valuable data. After careful evaluation, I believe it can be accepted for publication pending minor revisions to clarify certain points and improve readability. The authors are encouraged to address the specific comments provided to enhance the overall quality and clarity of the manuscript.

1. Please clearly state in the introduction that the DDR2-L610P variant could not be biochemically characterized due to solubility problems, to set appropriate expectations for the results section.
2. Briefly mention any limitations of using DDR1 as a reference model for DDR2, despite structural similarity.
3. The hypothesis—that DDR2 mutations relieve autoinhibition and enhance kinase activity—should be clearly articulated at the end of the introduction to guide the reader.
4. Briefly explain or break down long, complex sentences to improve clarity and readability. a sahi hai kya?
5. Clarify and expand how the increased DDR2 kinase activity caused by mutations leads to the clinical symptoms of Warburg-Cinotti syndrome to strengthen the biological relevance.
6. Consider adding a brief discussion on the limitations of using HEK293 cells as a model for DDR2 activity, as these cells may not fully recapitulate physiological conditions. OR Please briefly discuss the limitations of using HEK293 cells as a model system for DDR2 activity, including possible differences from endogenous expression contexts.
